# Heart Disease and Cerebrovascular Disease Mortality Trends, 2015–2025

**DOI:** 10.56786/PHWR.2026.19.41.1

**Published:** 2026-10-29

**Authors:** Yeonhee Yoo

**Affiliations:** Division of Chronic Disease Control, Department of Chronic Disease Prevention and Control, Korea Disease Control and Prevention Agency Cheongju Korea

In 2025, the mortality rates from heart disease and cerebrovascular disease in the Republic of Korea were 66.3 and 48.7 per 100,000 population, respectively. Compared with 2024, these rates increased by 0.6 and 0.5 deaths per 100,000, respectively. Over the decade, the mortality rate from heart disease has risen by 10.7 deaths per 100,000 (19.3%), with a greater increase in men (12.4) than in women (9.0), and heart disease mortality has been higher in men than in women since 2023. Cerebrovascular disease mortality remained higher in women, although the sex gap narrowed from 3.4 deaths per 100,000 in 2015 to 1.2 in 2025 (Figure 1).

## Supplementary Material



## Figures and Tables

**Figure 1:**
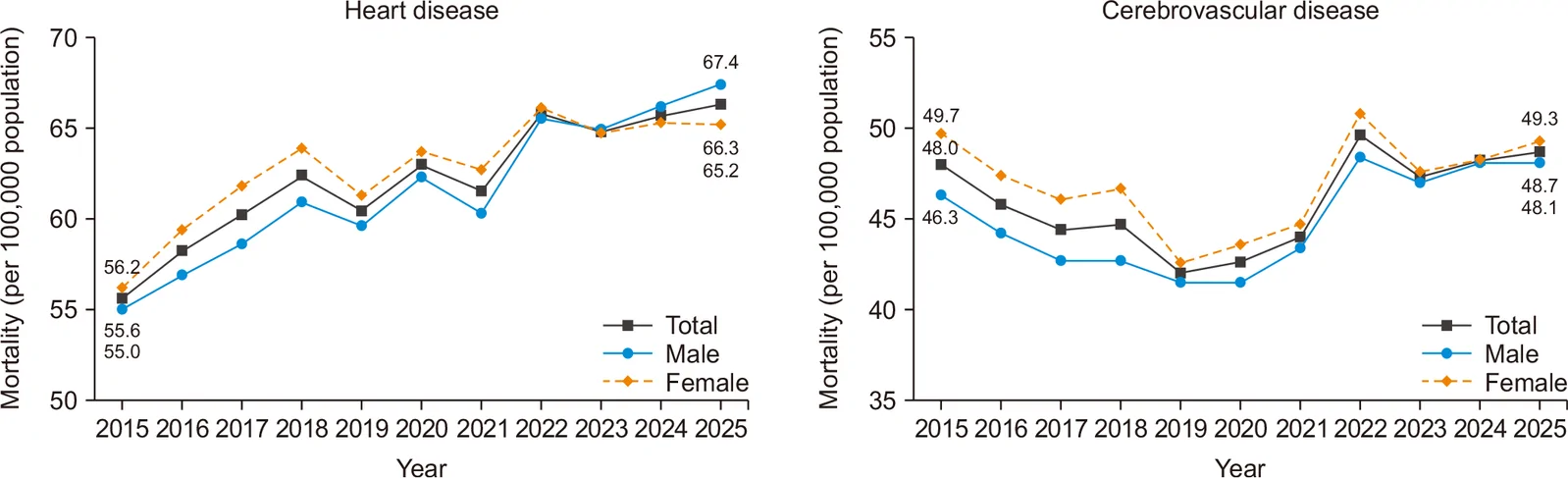
Heart disease and cerebrovascular disease mortality trends, 2015–2025 *Heart disease: I20-I51, cerebrovascular disease: I60-I69 ※Causes of death were classified according to the Korean Standard Classification of Diseases (KCD) **Cause-specific mortality rate=(number of deaths from a specific cause/mid-year population)×100,000

