# Supplementary material for: Heart Disease and Cerebrovascular Disease Mortality Trends, 2015–2025
Source: Public Health Wkly Rep. 2026 Oct 29;19(41):1735–6. doi: 10.56786/PHWR.2026.19.41.1 (PMC13720670; doi:10.56786/PHWR.2026.19.41.1)
Supplement: Translation

# Translation: 심뇌혈관질환 사망률 추이, 2015–2025년 – 사망원인통계

**Authors:** 연희 유

**Affiliations:** 질병관리청 만성질환관리국 만성질환관리과

2025년 우리나라의 심뇌혈관질환 사망률은 인구 10만 명당 심장질환 66.3명, 뇌혈관질환 48.7명으로 2024년 대비 각각 0.6명, 0.5명 증가하였다. 심장질환은 10년 전보다 10.7명(19.3%) 증가하였으며, 남자(12.4명)가 여자(9.0명)보다 크게 증가하여 2023년부터 남자의 사망률이 여자보다 높아졌다. 뇌혈관질환 사망률은 여자가 남자보다 높으나 성별 차이는 2015년 3.4명에서 2025년 1.2명으로 줄었다(그림 1).

## Figures and Tables

**그림 1:** 심장질환 및 뇌혈관질환 사망률 추이, 2015–2025년 *심장질환: I20-I51, 뇌혈관질환: I60-I69 ※한국표준질병·사인분류에 따라 사망원인을 집계 **사망원인별 사망률: (특정 사망원인에 의한 사망자 수/연앙인구)×100,000, 10만 명당 명
